# Relationship Between Coffee Consumption and Prevalence of Metabolic Syndrome Among Japanese Civil Servants

**DOI:** 10.2188/jea.JE20110068

**Published:** 2012-03-05

**Authors:** Hideo Matsuura, Kanae Mure, Nobuhiro Nishio, Naomi Kitano, Naoko Nagai, Tatsuya Takeshita

**Affiliations:** 1 Department of Public Health, Wakayama Medical University School of Medicine, Wakayama, Japan; 2 Wakayama City Public Health Center, Wakayama, Japan

**Keywords:** metabolic syndrome, coffee, cross-sectional study, Japan

## Abstract

**Background:**

Metabolic syndrome has become a major worldwide public health problem. We examined the relationship between coffee consumption and the prevalence of metabolic syndrome among Japanese civil servants.

**Methods:**

The study participants were 3284 employees (2335 men and 948 women) aged 20 to 65 years. Using data from their 2008 health checkup records, we analyzed the relationship between coffee consumption and the prevalence of metabolic syndrome. Metabolic syndrome was defined according to the Japanese criteria.

**Results:**

Metabolic syndrome was diagnosed in 374 of the 2335 men (16.0%) and 32 of the 948 women (3.4%). In univariate and multiple logistic regression analyses, the odds ratios (ORs) among men for the presence of metabolic syndrome were 0.79 (95% CI: 0.56–1.03) and 0.61 (0.39–0.95), respectively, among moderate (≥4 cups of coffee per day) coffee drinkers as compared with non-coffee drinkers. Among all components of metabolic syndrome, high blood pressure and high triglyceride level were inversely associated with moderate coffee consumption in men, after adjusting for age, body mass index, smoking status, drinking status, and exercise. However, in women, moderate coffee consumption was not significantly associated with the prevalence of metabolic syndrome or its components.

**Conclusions:**

Moderate coffee consumption was significantly associated with lower prevalence of metabolic syndrome in Japanese male civil servants.

## BACKGROUND

Metabolic syndrome is defined as a cluster of multiple risk factors, including central obesity, hypertension, dyslipidemia, and hyperglycemia,^1^^,^^2^ and is known to promote the development of cardiovascular diseases.^3^ Metabolic syndrome has become a major worldwide public health problem.^4^ Among Japanese aged 21 years or older, 25.3% of men and 10.6% of women meet the criteria for metabolic syndrome,^5^ and the prevalence of metabolic syndrome has been increasing in recent years, particularly among men.^5^ Thus, approaches to prevent it have been attempted in many workplaces and local governments in Japan.^6^^–^^8^

Coffee is one of the most widely consumed beverages in the world.^9^ Various effects of coffee consumption on the risks for diseases related to metabolic syndrome have been reported.^10^^–^^12^ During approximately the past decade, the associations between coffee consumption and many diseases, including hypertension, cardiovascular diseases, and cancer, have been studied, and its effects have been found to be harmful to health.^13^^,^^14^ However, several recent prospective cohort studies have indicated that coffee consumption lowers the risks of diabetes mellitus (DM), cardiovascular diseases, and cancer.^15^^,^^16^

The relationship between coffee consumption and metabolic syndrome has not been well investigated. To our knowledge, only a few studies have examined the relationship between coffee consumption and the risk of developing metabolic syndrome.^17^^,^^18^ One cross-sectional study showed that habitual coffee consumption was inversely associated with metabolic syndrome and its components among Japanese elderly adults.^17^ Another longitudinal study, which followed a Dutch population for 9 years, found no association between coffee consumption and the development of metabolic syndrome.^18^

In the present study, we investigated the association between coffee consumption and the prevalence of metabolic syndrome and its components among more than 3000 Japanese employees of a municipality.

## METHODS

### Participants

The participants in this study were 3873 employees (2550 men and 1323 women), aged 20 to 65 years, of a municipality in the Kansai area in western Japan. Of these 3873 participants, 3344 completed a self-administered questionnaire during an annual health examination in 2008 (response rate: 86.3%). After excluding participants who did not completely answer the relevant questions, the remaining 3283 participants (2335 men and 948 women) were included in the present analysis. This study was approved by the ethics committee of Wakayama Medical University (Approval No. 821).

### Questionnaire

The participants completed a self-administered questionnaire on their lifestyle, including coffee intake, alcohol drinking, smoking, and exercise.

There were 3 response choices for coffee consumption: do not drink, drink 1 to 3 cups/day, or drink 4 or more cups/day. We did not obtain information on the use of sugar and milk in coffee. There were also 3 choices for alcohol consumption: do not drink, sometimes drink, or drink every day. For smoking, there were 2 choices: do not smoke now or smoke now. There were 2 choices for exercise: do not exercise habitually or exercise habitually. Habitual exercise was defined as exercise with sweating at least twice per week for more than 1 year.^19^ We also asked about medication use for hypertension and high blood glucose: the 2 choices were yes or no.

### Laboratory testing

*Anthropometric measurements*: Height and weight were measured to calculate body mass index (BMI), ie, weight divided by the square of the height in meters. Waist circumference was measured at the umbilical level after normal expiration while participants stood unclothed.

*Blood assays*: Blood samples were drawn from the antecubital vein after overnight fasting. The serum was separated and centrifuged soon after blood coagulation. Plasma samples were collected in siliconized tubes containing sodium fluoride and were analyzed at 1 laboratory (Wakayama City Association, Wakayama prefecture, Japan) for blood measurement. Plasma glucose was measured using a glucose oxidase oxygen electrode method. Low-density lipoprotein (LDL) cholesterol, high-density lipoprotein (HDL) cholesterol, and triglyceride were determined using an autoanalyzer (TBA-C1600, Toshiba, Tokyo, Japan). LDL cholesterol and HDL cholesterol were measured using the direct method.

*Blood pressure measurement*: Blood pressure was measured by a trained staff member using a standard mercury sphygmomanometer placed around the right arm of a seated participant after at least 5 minutes of rest. When systolic blood pressure (SBP) was higher than 130 mm Hg or diastolic blood pressure (DBP) was higher than 85 mm Hg at the first measurement, blood pressure was measured again, and the second value was used in the analysis.

### Criteria for the diagnosis of metabolic syndrome

We defined metabolic syndrome according to Japanese criteria,^1^ namely, abdominal obesity (waist circumference: ≥85 cm in men or ≥90 cm in women) and at least 2 of the following components: (1) SBP 130 mm Hg or higher, DBP 85 mm Hg or higher, or use of antihypertensive medication, (2) serum triglyceride level 150 mg/dL or higher or HDL cholesterol level lower than 40 mg/dL, and (3) fasting plasma glucose (FPG) level 110 mg/dL or higher or use of antidiabetic medication. We did not obtain information on medical treatment for elevated triglyceride or low HDL cholesterol in the present study.

In an analysis of participants with metabolic syndrome who were not receiving medical treatment, we defined participants with abdominal obesity and 2 or more of the following components as having metabolic syndrome: (1) SBP 130 mm Hg or higher or DBP 85 mm Hg or higher, (2) serum triglyceride level 150 mg/dL or higher or HDL cholesterol level lower than 40 mg/dL, and (3) FPG 110 mg/dL or higher.

### Statistical analysis

Data were analyzed using SPSS version 15.0 (SPSS, Chicago, IL, USA). Differences in mean age and mean BMI across different levels of coffee consumption were tested in men and women using analysis of variance (ANOVA) with Bonferroni correction. Proportions of medical treatment, exercise, alcohol consumption, and cigarette smoking were tested using the χ^2^ test. Differences in the means for waist circumference, SBP, DBP, LDL cholesterol, HDL cholesterol, and logarithmic values of triglyceride and FPG, and proportions of those with metabolic syndrome, high blood pressure, high FPG, high triglyceride, low HDL, and high LDL across different levels of coffee consumption were statistically tested by analysis of covariance (ANCOVA). ANCOVA was used to estimate adjusted means and proportions across different levels of coffee consumption after adjustment for covariates. The estimated proportion of those with metabolic syndrome was adjusted for age and alcohol drinking, smoking, and exercise habits. The other estimated means and proportions were adjusted for age, BMI, and alcohol drinking, smoking, and exercise habits.

The association between coffee consumption and the prevalence of metabolic syndrome was evaluated by univariate and multivariate logistic regression analysis. In the multivariate models, we adjusted for age and alcohol drinking, smoking, and exercise habits. In all statistical tests, a *P* value less than 0.05 was considered significant.

## RESULTS

The characteristics of men and women by volume of coffee consumption are shown in Tables 1 and 2, respectively. We performed ANCOVA to estimate adjusted means and proportions and to evaluate differences. Among men, moderate coffee drinkers had lower mean SBP and DBP, higher mean LDL cholesterol, and lower proportions of metabolic syndrome, high blood pressure, and high triglyceride levels than did those who did not drink coffee. Among women, moderate coffee drinkers had a higher mean HDL cholesterol level than those who did not drink coffee.

**Table 1.** Characteristics of men by volume of coffee consumption

	Total	Coffee consumption		

		0 cups/day	1–3 cups/day	≥4 cups/day
*n*	2335	266	1658	411
Age (years)	46.4 ± 10.7^c^	44.6	46.6^e^	47.5^f^
Body mass index (kg/m^2^)	23.9 ± 3.2	23.7	23.9	24.0
Waist circumference (cm)	84.2 ± 9.0	84.1^h^	84.3	84.3
Systolic blood pressure (mm Hg)	124.0 ± 16.0	126.9^h^	125.2	122.3^f^
Diastolic blood pressure (mm Hg)	77.0 ± 11.0	78.8^h^	78.2	76.6^e^
Low-density lipoprotein cholesterol (mg/dL)	122.6 ± 29.7	119.9^h^	121.6	128.6^f^
High-density lipoprotein cholesterol (mg/dL)	57.9 ± 14.1	57.5^h^	58.1	57.8
Triglyceride (mg/dL)	107.0 (73.0, 161.0)^d^	114.8^h^	111.4	108.9
Fasting plasma glucose (mg/dL)	98.2 ± 22.8	98.6^h^	98.2	98.1

Metabolic syndrome (%)	16.0	18.9^i^	16.4	12.4^e^
High blood pressure (%)	43.9	49.7^h^	44.6	36.9^f^
Medical treatment for hypertension (%)	12.5	12.8	13.2	9.2
High fasting plasma glucose (%)	13.0	14.4^h^	12.8	12.9
Medical treatment for hyperglycemia (%)	3.9	4.5	5.1	6.1
High triglyceride^a^ (%)	28.3	30.6^h^	29.0	23.9^e^
Low high-density lipoprotein cholesterol^a^ (%)	5.7	7.7^h^	5.4	5.3
High low-density lipoprotein cholesterol (%)	26.5	23.7^h^	25.6	32.0
Exercise^b^ (%)				
No	67.2	67.7	66.3	70.6
Yes	32.8	32.3	33.7	29.4
Alcohol consumption (%)				
Non- or occasional drinkers	64.7	72.6	63.4	65.0
Habitual drinkers	35.3	27.4	36.6	35.0
Cigarette smoking (%)				
Non- or ex-smokers	56.3	77.1	56.4	42.6
Current smokers	43.7	22.9	43.6^g^	57.4^g^

^a^medical treatment for dyslipidemia was not assessed.

^b^exercise with sweating at least twice per week for more than a year.

^c^mean ± SD, ^d^median with 25th–75th quartile.

^e^*P* < 0.05, ^f^*P* < 0.01, ^g^*P* < 0.001 vs 0 cups/day for χ^2^ test, ANOVA, or ANCOVA.

^h^mean adjusted for age, BMI, alcohol drinking, smoking, and exercise in the 3 coffee consumption groups.

^i^mean adjusted for age, alcohol drinking, smoking, and exercise by the 3 coffee consumption groups.

**Table 2.** Characteristics of women by volume of coffee consumption

	Total	Coffee consumption		

		0 cups/day	1–3 cups/day	≥4 cups/day
*n*	948	169	678	101
Age (years)	46.4 ± 9.4^c^	41.9	47.2^g^	48.8^g^
Body mass index (kg/m^2^)	21.9 ± 3.7	22.0	22.0	21.6
Waist circumference (cm)	77.1 ± 10.3	77.1^h^	77.0	77.4
Systolic blood pressure (mm Hg)	118.0 ± 16.0	119.1^h^	118.0	118.4
Diastolic blood pressure (mm Hg)	71.0 ± 10.0	72.8^h^	71.5	71.4
Low-density lipoprotein cholesterol (mg/dL)	121.0 ± 33.1	116.8^h^	121.5	125.1
High-density lipoprotein cholesterol (mg/dL)	72.0 ± 15.0	69.0^h^	72.5	73.7^g^
Triglyceride (mg/dL)	67.0 (51.0, 92.0)^d^	71.3^h^	71.0	68.9
Fasting plasma glucose (mg/dL)	91.2 ± 12.0	92.8^h^	92.0	90.6

Metabolic syndrome (%)	3.4	4.1^i^	3.4	2.2
High blood pressure (%)	27.4	28.0^h^	28.0	22.5
Medical treatment for hypertension (%)	6.9	5.9	7.4	5.0
High fasting plasma glucose (%)	5.6	7.2^h^	5.3	4.8
Medical treatment for hyperglycemia (%)	1.6	2.4	5.0	4.0
High triglyceride^a^ (%)	7.1	7.0^h^	7.3	5.9
Low high-density lipoprotein cholesterol^a^ (%)	0.7	1.0^h^	0.8	0.0
High low-density lipoprotein cholesterol (%)	25.7	23.3^h^	25.6	30.9
Exercise^b^ (%)				
No	86.0	92.9	84.4	85.1
Yes	14.0	7.1	15.6^f^	14.9
Alcohol consumption (%)				
Non- or occasional drinkers	90.4	92.9	90.0	89.1
Habitual drinkers	9.6	7.1	10.0	10.9
Cigarette smoking (%)				
Non- or ex-smokers	93.8	94.1	95.1	84.2
Current smokers	6.2	5.9	4.9	15.8^e^

^a^medical treatment for dyslipidemia was not assessed.

^b^exercise with sweating at least twice per week for more than a year.

^c^mean ± SD, ^d^median with 25th–75th quartile.

^e^*P* < 0.05, ^f^*P* < 0.01, ^g^*P* < 0.001 vs 0 cups/day for χ^2^ test, ANOVA, or ANCOVA.

^h^mean adjusted for age, BMI, alcohol drinking, smoking, and exercise in the 3 coffee consumption groups.

^i^mean adjusted for age, alcohol drinking, smoking, and exercise in the 3 coffee consumption groups.

The relationship between coffee consumption and the prevalence of metabolic syndrome was evaluated in men and women using univariate and multivariate logistic regression analyses adjusted for age, alcohol consumption, exercise, and smoking status (Table 3).

**Table 3.** Logistic regression analysis of the associations between coffee consumption and prevalence of metabolic syndrome by sex

(1) Men							

Variables		Univariate			Multivariate^b^		
	
		OR^c^	95% CI^c^	*P* value	OR	95% CI	*P* value
Including those receiving medical treatment^a^ (*n* = 2335)							
Coffee consumption	1–3 cups/day	1.12	0.88–1.44	0.355	0.85	0.59–1.20	0.351
	≥4 cups/day	0.79	0.56–1.03	0.080	0.61	0.39–0.95	0.028

Excluding those receiving medical treatment^a^ (*n* = 1992)							
Coffee consumption	1–3 cups/day	1.25	0.93–1.69	0.145	0.95	0.62–1.46	0.810
	≥4 cups/day	0.70	0.48–1.03	0.068	0.60	0.35–1.03	0.064

(2) Women							

Variables		Univariate			Multivariate^b^		
	
		OR^c^	95% CI^c^	*P* value	OR	95% CI	*P* value

Including those receiving medical treatment^a^ (*n* = 948)							
Coffee consumption	1–3 cups/day	1.02	0.47–2.23	0.964	0.74	0.29–1.90	0.528
	≥4 cups/day	0.86	0.26–2.89	0.812	0.48	0.11–2.09	0.327

Excluding those receiving medical treatment^a^ (*n* = 874)							
Coffee consumption	1–3 cups/day	0.46	0.17–1.28	0.136	0.32	0.10–1.02	0.054
	≥4 cups/day	1.25	0.28–5.63	0.770	0.38	0.06–2.29	0.291

^a^medical treatment for hypertension and hyperglycemia.

^b^adjusted for age, alcohol drinking, smoking, and exercise.

^c^OR, odds ratio; CI, confidence interval.

Among men, including those receiving medical treatment, the odds ratio (OR) of metabolic syndrome associated with moderate coffee consumption as compared with no coffee consumption was 0.79 (95% CI: 0.56–1.03) in the univariate model. In the multivariate model, the OR was 0.61 (0.39–0.95) for moderate coffee consumption. Likewise, after excluding those receiving medical treatment, the OR for moderate coffee consumption was 0.70 (0.48–1.03) among men in the univariate model. In the multivariate model, the OR was 0.60 (0.35–1.03) for moderate coffee consumption.

Among women, including those receiving medical treatment, coffee consumption was not significantly associated with the prevalence of metabolic syndrome in either the univariate or multivariate model. Likewise, among women not receiving medical treatment, coffee consumption was not significantly associated with the prevalence of metabolic syndrome in the univariate or multivariate model.

## DISCUSSION

The present study examined the relationship between coffee consumption and the prevalence of metabolic syndrome and its components in a cross-sectional study of Japanese civil servants. The main finding is that moderate coffee consumption (≥4 cups/day) was significantly associated with lower prevalence of metabolic syndrome in men. Moreover, components of metabolic syndrome, ie, hypertension and high triglycerides, were significantly inversely associated with moderate coffee consumption among men. However, among women, moderate coffee consumption was not significantly associated with the prevalence of metabolic syndrome or its component. It is likely that the small sample size of women with metabolic syndrome precluded detailed analyses in women.

To our knowledge, the relationship between coffee consumption and metabolic syndrome has not yet been well investigated among younger or middle-aged participants. Hino et al^17^ reported that coffee consumption was inversely associated with metabolic syndrome and all its components in older local residents in Japan. The present findings are in line with their results. However, Driessen et al^18^ reported no relation between coffee consumption and the development of metabolic syndrome or its components in a Dutch population. Only a few other studies have investigated the associations between coffee consumption and metabolic syndrome.

The tendency toward an association between coffee consumption and the prevalence of metabolic syndrome remained when we excluded participants receiving medical treatment. Therefore, it is not likely that medical treatment significantly influenced the observed association.

Some studies have indicated that coffee consumption increases blood pressure.^12^^,^^20^ However, a recent dose-response meta-analysis showed an inverse J-shaped curve, with hypertension risk increasing up to 3 cups/day and decreasing with higher intake.^21^ Some Japanese studies have shown an inverse association between moderate coffee consumption and blood pressure.^17^^,^^22^^,^^23^ The present results are consistent with these findings.

Many recent epidemiologic studies suggest that regular coffee intake has beneficial effects on risk of DM.^24^^,^^25^ In Japan, a prospective study reported that consuming 3 or more cups of coffee per day was inversely associated with DM as compared with a reference group (0–1 cups of coffee per day).^26^ In the present study, FPG and moderate coffee consumption (≥4 cups/day) were not correlated among men and women.

In the present study, moderate coffee consumption was significantly inversely associated with the proportion of those with high triglyceride levels in men. Hino et al^17^ reported that coffee consumption was inversely associated with the proportion of those with high triglyceride levels but not with the proportion of those with HDL cholesterol. Driessen et al,^18^ on the other hand, reported no association of coffee consumption with HDL cholesterol or triglyceride levels. Our results are consistent with those of Hino et al.^17^

Our study also showed a positive association between LDL cholesterol level and moderate coffee consumption in men. A meta-analysis of randomized controlled trials of the association between coffee consumption and serum lipids showed that consuming 6 cups/day was associated with increased LDL cholesterol, although trials with filtered coffee showed very little increase in serum cholesterol.^27^ The cholesterol-raising factors have been shown to be the diterpenes cafestol and kahweol,^28^ which are removed by filtering.^29^ Miyake et al^30^ suggested that instant coffee, but not brewed coffee, is associated with increased LDL cholesterol. Further comprehensive studies are required to examine the relation between coffee consumption and serum lipids, including the type of preparation method used.

This study has several limitations. First, its cross-sectional design does not allow us to make inferences regarding the causality of the observed associations. However, it is likely that coffee drinking has a preventive effect against metabolic syndrome because the habit of coffee drinking likely continues for years. On the other hand, it is also possible that people with abnormal health check-up results, or those who are on a medication, abstain from coffee drinking, especially considering the fact that earlier studies indicated that coffee had rather harmful effects on health. Hence, prospective studies are needed to confirm the causal relationships between coffee consumption and the prevalence of metabolic syndrome.

Second, our study could not analyze the dose-response relationship in detail because the questionnaire on coffee consumption had only 3 choices (never, 1–3 cups/day, ≥4 cups/day). We need to obtain more detailed information on amount of coffee consumed, methods of coffee preparation (eg, filtered or instant), and use of coffee additives (eg, sugar, milk, and cream).^31^

Third, we did not obtain information on medical treatment for either elevated triglyceride or low HDL cholesterol in relation to the diagnosis of metabolic syndrome. Therefore, the number of those with dyslipidemia would be smaller than the true number in this study, which might have somewhat influenced the present results.

Fourth, the participation rates among participants 40 years or older and those younger than 40 years were 97.4% (2315/2376) and 61% (590/968), respectively. Selection bias was likely minimal among the former because of the high participation rate, although it might have influenced the findings to some extent among the latter.

Finally, all participants were civil servants. The proportion of civil service employees in Japan is around 3.5%, and they represent workers with higher than average job security and income; they also enjoy a healthier lifestyle in comparison to other segments of society.^32^ Therefore, caution should be used in applying the present results to other populations.

The present study showed a significant relation between coffee consumption and metabolic syndrome in men. Further prospective cohort studies are needed to clarify the relationship between coffee consumption and the prevalence of diseases related to metabolic syndrome. A recent meta-analysis of 21 prospective cohort studies suggested that habitual moderate coffee drinking was associated with a lower risk of coronary heart disease (CHD).^33^ In the present study, while moderate coffee consumption was inversely associated with hypertension and high triglyceride levels, it was positively associated with high LDL cholesterol levels. Elevated LDL cholesterol level is strongly related to increased risk of CHD.^30^ Therefore, it remains to be determined how long-term coffee consumption influences the risk for CHD, especially with regard to methods of coffee preparation and the use of coffee additives. Regarding cancer, the results of meta-analyses suggest an inverse association of coffee consumption with colorectal and liver cancer.^15^^,^^34^ Further large-scale studies are required to clarify the effects of long-term coffee consumption on the risks of CHD and cancer.

In conclusion, we found that coffee consumption (≥4 cups/day) was significantly associated with lower prevalences of metabolic syndrome (after adjustment for age and other important lifestyle factors) and its components (after adjustment for age, BMI, and other important lifestyle factors) in men. We plan to further investigate the role of long-term coffee consumption in preventing health outcomes related to metabolic syndrome, including cardiovascular diseases and cancer, in a follow-up study that will assess the present participants for the duration of their employment with the municipality.
